# USP25 attenuates anti-GBM nephritis in mice by negative feedback regulation of Th17 cell differentiation

**DOI:** 10.1080/0886022X.2024.2338932

**Published:** 2024-04-14

**Authors:** Ranran Xu, Fei Huang, Qingquan Liu, Yongman Lv, Liu Hu, Qian Zhang

**Affiliations:** a Department of Geriatrics, Tongji Hospital, Tongji Medical College, Huazhong University of Science and Technology, Wuhan, P. R. China; b Department of General Medicine, Tongji Hospital, Tongji Medical College, Huazhong University of Science and Technology, Wuhan, P. R. China; c Department of Nephrology, Tongji Hospital, Tongji Medical College, Huazhong University of Science and Technology, Wuhan, P. R. China; d Health Management Centre, Tongji Hospital, Tongji Medical College, Huazhong University of Science and Technology, Wuhan, P. R. China

**Keywords:** USP25, anti-GBM GN, Th17 cells, RORγt

## Abstract

**Purpose:**

This study aimed to elucidate the role of USP25 in a mouse model of anti-glomerular basement membrane glomerulonephritis (anti-GBM GN).

**Methods:**

USP25-deficient anti-GBM GN mice were generated, and their nephritis progression was monitored. Naïve CD4+ T cells were isolated from spleen lymphocytes and stimulated to differentiate into Th1, Th2, and Th17 cells. This approach was used to investigate the impact of USP25 on CD4+ T lymphocyte differentiation *in vitro*. Furthermore, changes in USP25 expression were monitored during Th17 differentiation, both *in vivo* and *in vitro*.

**Results:**

USP25−/− mice with anti-GBM GN exhibited accelerated renal function deterioration, increased infiltration of Th1 and Th17 cells, and elevated RORγt transcription. *In vitro* experiments demonstrated that USP25−/− CD4+ T lymphocytes had a higher proportion for Th17 cell differentiation and exhibited higher RORγt levels upon stimulation. Wild-type mice with anti-GBM GN showed higher USP25 levels compared to healthy mice, and a positive correlation was observed between USP25 levels and Th17 cell counts. Similar trends were observed *in vitro*.

**Conclusion:**

USP25 plays a crucial role in mitigating renal histopathological and functional damage during anti-GBM GN in mice. This protective effect is primarily attributed to USP25’s ability to inhibit the differentiation of naïve CD4+ T cells into Th17 cells. The underlying mechanism may involve the downregulation of RORγt. Additionally, during increased inflammatory responses or Th17 cell differentiation, USP25 expression is activated, forming a negative feedback regulatory loop that attenuates immune activation.

## Introduction

Anti-glomerular basement membrane (GBM) disease is an autoimmune disorder that specifically targets the kidney, manifesting as glomerular injury mediated by antibodies, and linear immune deposits along the GBM. The typical manifestation is crescentic glomerulonephritis (CGN), often leading to acute renal failure and occasionally complicated by pulmonary hemorrhage, known as Goodpasture’s syndrome [1]. Untreated, this condition can rapidly progress to life-threatening end-stage renal failure within weeks of its onset [2–5].

The pathological features of CGN involve the compromise of the capillary wall integrity and the infiltration of T cells, macrophages, and plasma proteins into the renal sacs. Among these cells, CD4+ T cells play a pivotal role in mediating kidney injury and repair [6]. Numerous studies have demonstrated that the infiltration and activation of Th1 and Th17 cells are intricately linked to crescentic glomerulonephritis [7]: (1) The absence of IL-12 [8] attenuates CGN damage, similar to the blocking of Th1 cytokines, while the administration of IL-12 exacerbates the condition [9]. (2) The utilization of anti-IFNγ (a Th1 cytokine) antibody [8] and the IFNγ^−/−^ mice both result in reduced crescent formation [10,11]. (3) Kidney tissue damage is significantly diminished in IL-17F-deficient mice, compared to wild-type mice [12]. (4) When wild-type mice with nephritis are treated with anti-IL-17F neutralizing antibodies, histological damage scores are significantly reduced compared to those treated with isotype antibody [12]. (5) IL-17F-deficient lupus mice exhibit reduced mortality and albuminuria compared to regular lupus mice [13]. (6) RORγt, a crucial transcription factor in Th17-cell development, has been shown to promote CGN [14]. Therefore, targeting the immune response mediated by Th1 and Th17 cells holds promise as a therapeutic strategy to mitigate the progression of CGN [15,16].

Recently, the reversible ubiquitination of cell signaling molecules has emerged as a crucial mechanism for cellular responses to extracellular stimuli. This highly regulated dynamic process involves the intricate interplay between E3 ubiquitinating ligase and deubiquitinating enzymes (DUBs) [17]. Recent reports have indicated that the ubiquitination of the TAK1-TAB1 complex plays a pivotal role in regulating Th17 cell differentiation [18]. Similarly, ubiquitination of TRAF5 and TRAF6 has been shown to impact IL17-mediated signaling and inflammatory responses [19]. Furthermore, ubiquitin activating enzymes are thought to maintain tolerance in Th1 immune responses by suppressing NF-κB activation [20]. These findings underscore the significance of ubiquitination in regulating cellular processes and immune responses. Ubiquitin-specific protease (USP), a member of the DUB family, plays a crucial role in removing ubiquitin molecules from large proteins [21]. USP25, a significant member of the USP subfamily, exhibits high homology and is subject to strict regulatory controls [22]. Its upregulation has been observed in various tumors [23], neurodegenerative diseases [24–26], and immune system diseases [27,28]. In a study comparing wild-type and USP25-deficient mice, the induction of experimental autoimmune encephalomyelitis (EAE) revealed a significant exacerbation of EAE pathology in USP25−/− mice. Notably, the expression of pro-inflammatory factors in the central nervous system of USP25−/− mice was significantly elevated compared to wild-type mice, including IL-6, CXC chemokine ligand 1 (CXCL1), and CC chemokine ligand 20 (CCL20) [29]. These findings suggest that USP25 suppresses the development of EAE and regulates immune responses [29]. However, the role of USP25 in modulating the progression of anti-glomerular basement membrane glomerulonephritis (anti-GBM GN) remains elusive. In this study, an experimental model of anti-GBM GN was established in both wild-type and USP25-deficient mice. The aim was to investigate the association between USP25 and anti-GBM GN, as well as the link between USP25 and the differentiation of CD4+ helper T cell subsets, particularly Th17 cells. The findings of this study will offer a theoretical foundation for the targeted treatment of USP25 against anti-GBM GN.

## Materials and methods

### Mice

The animals utilized in this experiment were bred in the SPF Animal Laboratory, Animal Research Center, Tongji Medical College, Huazhong University of Science and Technology, to ensure optimal conditions for the study. USP25−/− mice were purchased from Beijing Vital River Laboratory Animal Technology Co., Ltd. The gene knockout strategy employed in this study involved the design of two sgRNAs targeting exon 3 of the USP25 allele in mice. This approach aimed to induce DNA double-strand breaks, resulting in a frameshift mutation in the USP25 gene upon NHEJ repair. Mouse embryonic cells harboring these USP25 mutations were then microinjected into blastocysts to generate chimeric mice.

Mice were genotyped through PCR analysis of DNA extracted from tail tissue samples (primers: USP25-GT-F1 TAGCAGTAGCCTTCCTCACTGC, USP25-GT-R1 CTCGCTCAAGCTTTACTGTGCC). USP25± mice were crossed to generate age- and sex-matched littermates of USP25+/+ and USP25−/− mice. Female MRL/MPJ mice and MRL/MPJ-*Fas^lpr^*/J mice, aged 4–5 weeks and weighing 10–15 grams, were purchased from Jackson Laboratories in the United States (agent: Beijing Chengtian Biotechnology Co., LTD., China). All animal experiments were approved by the Animal Care and Use Committee of Tongji Hospital, Tongji Medical College, Huazhong University of Science and Technology, ensuring compliance with ethical standards and regulations governing animal research.

### Induction of anti-GBM GN mice

Anti-GBM GN was induced in both C57BL/6 mice and USP25−/− mice following a specific protocol. Briefly, mice were intraperitoneally injected with 0.2 mL of sheep IgG (I5131, Sigma-Aldrich; 0.02 mg/g) mixed with an equal volume of complete Freund’s adjuvant (344289, Sigma-Aldrich). Ten days later, the experimental group received an intravenous injection of sheep anti-mouse serum (10 µl/g, generously provided by Prof. Fan He and Dr. Yi Yang, purchased from Elabscience-Wuhan) through the tail vein, while the control group was injected with normal sheep serum. Mice were euthanized, and experimental specimens were collected on the 7th, 14th, and 21st days following the tail vein injection.

### Processing specimen

Immediately following euthanasia at the designated time points, the kidneys were collected from the mice. Kidney tissue paraffin sections were prepared and stained with periodic acid-Schiff (PAS) for histological analysis. Additionally, kidney tissues from the same region were embedded in OCT and frozen sections were prepared. Direct immunofluorescence staining was performed by adding Alexa Fluor 594 monkey anti-sheep IgG (A-11016, Thermo Fisher Scientific; 1:100) and Alexa Fluor 594 monkey anti-mouse IgG (R37115, Thermo Fisher Scientific; 1:100).

Blood samples were collected from the mice. These samples were then tested using a serum creatinine (Scr) kit (ABIN577685, Biovision Inc., Milpitas) and a blood urea nitrogen (BUN) test (BK-600, Biobase Inc.) to assess kidney function.

Spleen single cell suspensions were prepared according to a previously described protocol [30]. CD4 T cells were then isolated, and multicolor flow cytometric analysis was performed to determine specific cell proportions.

For real-time PCR and ELISA, total RNA was extracted from kidney tissue using Trizol (R411-01, Vazyme). cDNA was synthesized using 1 μg of RNA. Real-time PCR was conducted with SYBR Green mix (Q121-02, Vazyme). Data were normalized to the expression of GAPDH. The primers used are provided in the Supplementary Materials. IFN-γ, IL-4, and IL-17A levels in the samples were measured using corresponding ELISA kits (CSB-E04578m, CSB-E04634m, CSB-E04608m, Cusabio). For Western blotting, the experimental protocol was followed as described previously [30]. Antibodies used were rabbit USP25 (2B6A2, Proteintech; 1:250) and rabbit anti-β-actin (#4967S, CST; 1:4,000).

### Isolation and directed differentiation of spleen CD4 T cells

Previously established protocols were utilized to isolate spleen cells and generate a single-cell suspension, from which CD4+ T cells were isolated [30]. To induce Th1 cell differentiation, the following cytokines and antibodies were used: IL-2 (100 ng/ml), IL-12 (10 ng/ml), and anti-IL-4 (10 μg/ml). For Th2 cell differentiation, the conditions included IL-2 (100 ng/ml), IL-4 (20 ng/ml), anti-IFN-γ (10 μg/ml), and anti-IL-12 (10 μg/ml). To promote Th17 cell differentiation, the media was supplemented with IL-2 (100 ng/ml), TGF-β1 (5 ng/ml), IL-6 (10 ng/ml), anti-IFN-γ (10 μg/ml), and anti-IL-4 (10 μg/ml). Finally, for Treg cell differentiation, the cells were cultured in the presence of IL-2 (100 ng/ml), anti-IFN-γ (10 μg/ml), and anti-IL-4 (10 μg/ml).

## Results

### *USP25*−*/*− *mice*

To assess the impact of USP25 on immune progression in anti-GBM GN mice, USP25−/− mice were obtained from a laboratory animal company using knockout strategies detailed in the Materials and Methods section. These knockout mice exhibited normal growth and survival, with bright fur, good appetite, regular urination and defecation, and mental alertness and activity. No significant differences were observed in body weight between USP25−/− mice and their wild-type littermates at 4–8 weeks of age. Furthermore, at 8 weeks of age, there were no notable disparities in renal function (Scr, BUN), splenic Th1, Th2, and Th17 lymphocyte levels, serum inflammatory cytokines (IFN, IL-4, and IL-17A), or the characteristic transcription factors (T-bet, GATA3, FOXP3, and RORgt) of kidney Th1, Th2, Treg, and Th17 cells between USP25−/− mice and wild-type mice (Supplementary Figures S1–S4). These findings suggest that USP25 is dispensable for mouse growth and development and does not significantly impact immune cell populations or serum inflammatory cytokines in these mice.

### Knockout USP25 exacerbated the renal injury in anti-GBM GN

**Figure 1.**
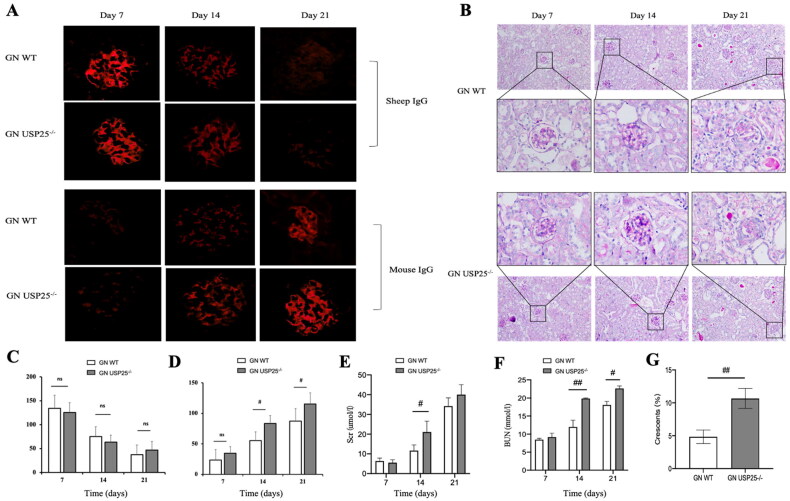
Knockout USP25 aggravated the renal injury in anti-GBM GN: (A) Representative photographs of the deposition of sheep IgG and mouse IgG in the kidneys of WT anti-GBM GN mice and USP25^−/−^ anti-GBM GN mice; (B) pathological changes in renal tissues in wild anti-GBM GN mice and USP25^−/−^ anti-GBM GN mice; (C and D) quantification deposition of sheep IgG and mouse IgG; (E and F) Scr (E) and BUN (F) of USP25^−/−^ anti-GBM GN mice and wild anti-GBM GN mice; (G) the proportion of crescent formation at the day 21. Anti-GBM GN: anti-glomerular basement membrane glomerulonephritis; Scr: serum creatinine; BUN: blood urea nitrogen; WT: wild type; bars represent means ± SEM; *n* = 5/group; ^#^*p* < 0.05; ^##^*p* < 0.01.

Wild-type mice and USP25−/− mice were simultaneously induced to develop anti-GBM GN. Immunohistochemical staining of kidney tissue sections revealed that the fluorescence intensity of mouse IgG was stronger in USP25−/− mice compared to wild-type mice at the same time point. Notably, there was no significant difference in the distribution of sheep IgG in the glomeruli between the two groups (Figure 1(A,C,D). Histopathological examination of renal tissues from wild-type GN mice and USP25−/− GN mice demonstrated more severe renal injury in USP25−/− mice, characterized by increased crescent formation and exacerbated interstitial damage (Figure 1(B,G)). To assess renal function, kidney function tests were performed on the 7th, 14th, and 21st days. The results demonstrated that USP25−/− mice experienced a more rapid decline in renal function compared to their wild-type counterparts (Figure 1(E,F)). Collectively, these findings suggest that USP25 knockout exaggerates kidney injury and accelerates renal deterioration in anti-GBM GN mice.

### Knockout of USP25 increased Th17 cells in vivo and in vitro

**Figure 2.**
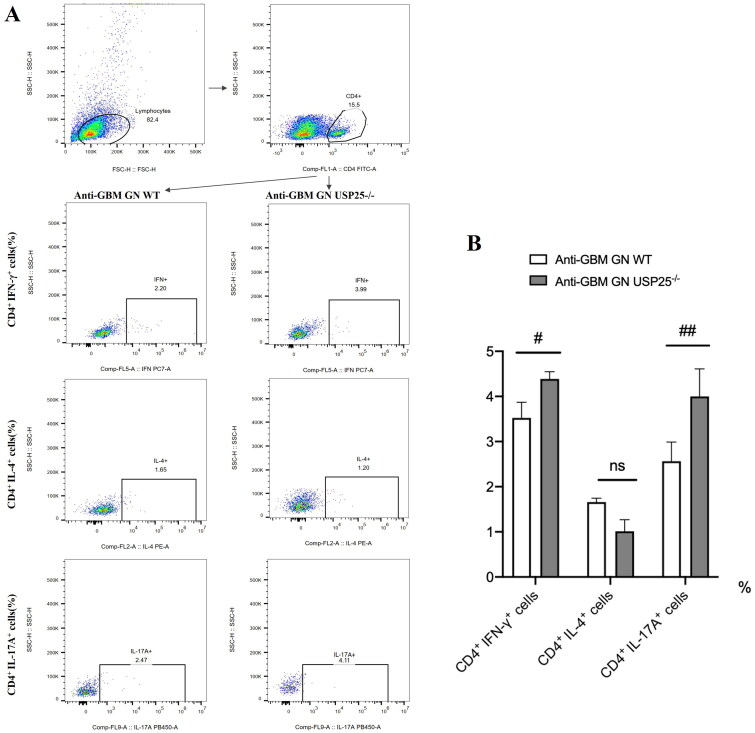
Knockout of USP25 increased Th17 cells *in vivo*: (A and B), proportion of spleen Th1, Th2, Th17 in wild mice and USP25^−/−^ mice 14 days after establishment of the anti-GBM GN; Anti-GBM GN: anti-glomerular basement membrane glomerulonephritis; WT: wild type; bars represent means ± SEM; *n* = 3–5 each group; ^#^*p* < 0.05; ^##^*p* < 0.01.

Flow cytometry was utilized to quantify the proportions of splenic Th1, Th2, and Th17 cells (key immune cells implicated in anti-GBM GN) in both wild-type and USP25−/− mice 14 days post-induction of the anti-GBM GN model (Figure 2(A)). The findings (Figure 2(B)) revealed that the absence of USP25 led to a higher prevalence of Th1 and Th17 cells in the spleen compared to wild-type mice, while there was no notable difference in the proportion of Th2 lymphocytes.

**Figure 3.**
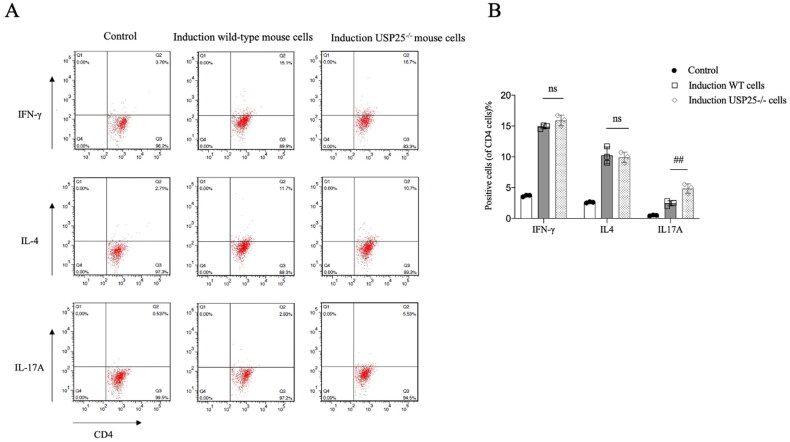
Percentage of positive cells after CD4+ T cell induced *in vitro* of USP25^−/−^ mice and WT mice. WT: wild type; bars represent means ± SEM; *n* = 3–5 each group; ns: none significance; ^##^*p* < 0.01.

To investigate the impact of USP25 on the differentiation of Th1, Th2, and Th17 cells *in vitro*, we isolated spleen lymphocytes from both wild-type and USP25−/− mice and prepared cell suspensions for the purpose of isolating CD4+ T lymphocytes and inducing their differentiation. As depicted in Figure 3(A,B), the absence of USP25 had minimal influence on the differentiation of Th1 and Th2 cells. However, a notable increase in the proportion of Th17 cells was observed.

### USP25 inhibits transcription of RORγt in vivo and in vitro

**Figure 4.**
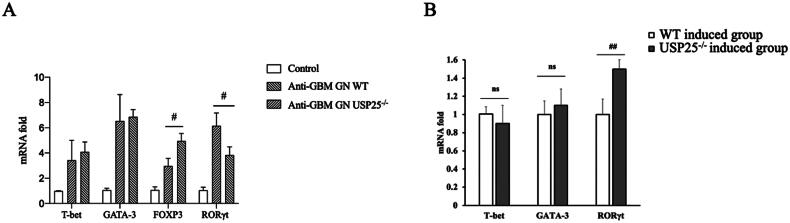
Knockout of USP25 increase transcription of RORγt *in vitro* (A) and *in vitro* (B): (A) RORγt mRNA levels in the kidney of USP25^−/−^ mice and wild mice at 14 days at the anti-GBM GN model; (B) CD4 T cells were isolated from the spleen of wild mice and USP25^−/−^ mice, and then induced to Th1, Th2 and Th17 cells. The mRNA levels of RORγt were measured after cell differentiation. Anti-GBM GN, anti-glomerular basement membrane glomerulonephritis; WT: wild type; bars represent means ± SEM; *n* = 3–4 each group; ^#^*p* < 0.05; ^##^*p* < 0.01.

To further elucidate the role of USP25 in Th17 cell differentiation, we examined RORγt, a key transcription factor specific to Th17 cells, both *in vivo* and *in vitro. In vivo*, we compared the RORγt transcription levels in USP25−/− mice and wild-type mice 14 days after establishing the anti-GBM GN model. The results revealed that the expression of RORγt in the kidneys of USP25−/− mice was significantly higher than that observed in wild-type mice (Figure 4(A)). To complement our *in vivo* observations, we conducted an *in vitro* study in which CD4+ T cells were isolated from the spleens of both wild-type and USP25−/− mice. After activating and proliferating these cells, we measured the mRNA levels of characteristic transcription factors for Th1, Th2, and Th17 cells. Consistent with the vivo results, we found that the RORγt mRNA level was significantly higher in USP25-defective cells compared to wild-type cells (Figure 4(B)).

### USP25 levels are positively correlated with Th17 levels in vivo and in vitro

**Figure 5.**
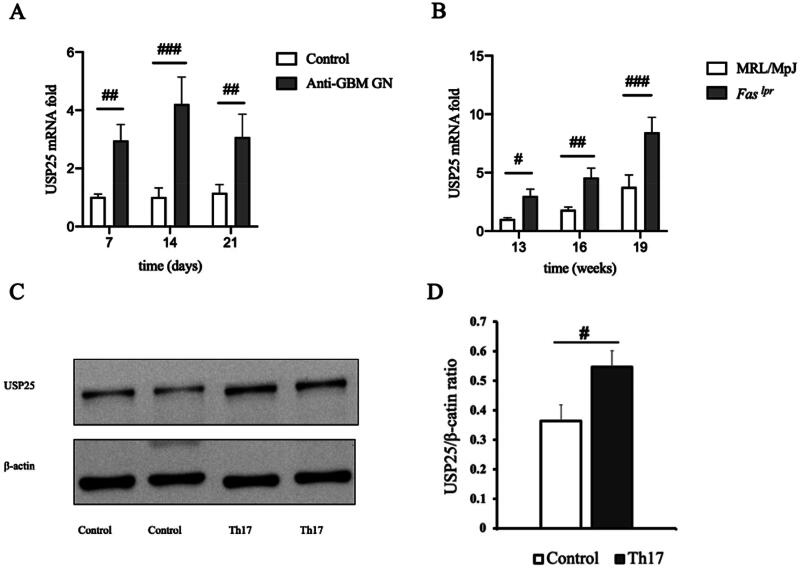
USP25 increased after establishment of anti-GBM GN or Th17 cell differentiation. (A and B) The mRNA expression of USP25 in C57BL/6 mice with anti-GBM GN (A) and spontaneous lupus erythematosus mice (B) at different points in time. (C and D) Expression of USP25 after stimulating CD4+ T cells into Th17 cells *in vitro*. Anti-GBM GN: anti-glomerular basement membrane glomerulonephritis; *Fas^lpr^*, a mouse model of spontaneous lupus erythematosus; MRL/MpJ is control mouse of *Fas^lpr^*; bar, means ± SEM; ^#^*p* < 0.05; ^##^*p* < 0.01; ^###^*p* < 0.001.

To gain a deeper understanding of the dynamic expression of USP25 in the development of anti-GBM GN, we employed RT-PCR to measure USP25 levels in kidney tissue from wild mice with anti-GBM GN. The results revealed that USP25 expression in the model group was elevated compared to the control group, exhibiting a pattern of initial increase followed by a decrease (Figure 5(A)). When comparing these results with the dynamics of immune cell monitoring in wild mice with anti-GBM GN, it was found that the level of Th17 cells fluctuated in synchrony with USP25 levels. Additionally, we explored USP25 expression in kidney tissue from a mouse model of spontaneous systemic lupus erythematosus (MRL/MpJ-*Fas^lpr^*/J mice). The analysis indicated an upward trend in USP25 expression in *Fas^lpr^* mice aged 13–19 weeks (Figure 5(B)).

*In vitro*, we employed the aforementioned method to induce and differentiate Th17 cells while monitoring alterations in USP25 expression. It was found that USP25 expression underwent an elevation following the induction of Th17 cell differentiation (Figure 5(C,D)).

## Discussion

Our experimental findings indicate that USP25 plays a significant role in alleviating renal histopathological and functional damage during anti-GBM GN. The mechanism behind this protective effect is primarily linked to USP25’s ability to inhibit the differentiation of naive CD4+ T cells into Th17 cells. Both *in vivo* and *in vitro* experiments have demonstrated that USP25 suppresses the expression of RORγt, a key transcription factor for Th17 cell differentiation. Interestingly, as the inflammatory response intensifies or Th17 cell differentiation increases, USP25 expression is upregulated, suggesting the existence of a negative feedback regulatory pathway. This pathway serves to attenuate Th17 cell differentiation and immune activation, thereby mitigating renal damage in anti-GBM GN. In summary, our studies highlight the protective role of USP25 in renal pathophysiology and its potential as a therapeutic target for the treatment of immune-mediated kidney diseases.

USP, as a deubiquitylation enzyme, has garnered significant attention and research in recent years due to its ability to remove ubiquitin molecules from proteins, thereby altering their fate and function [31]. One particular member of this family, USP25, has been implicated in various immunological processes. Studies have shown that USP25 plays a crucial role in the innate antiviral immune response [32,33]. Mice lacking USP25 exhibit increased susceptibility to lipopolysaccharide (LPS)-induced septic shock, which is associated with elevated production of pro-inflammatory cytokines and reduced interferon-α production [32]. This suggests that USP25 functions to modulate the inflammatory response and prevent excessive immune activation. In the context of experimental autoimmune encephalomyelitis (EAE), a mouse model of multiple sclerosis, USP25-deficient mice display more severe EAE pathology compared to wild-type mice. The number of macrophages and neutrophils infiltrating into the central nervous system (CNS) increased in USP25−/− mice, and the expression of pro-inflammatory genes (including Il6, Cxcl1, and Ccl20) in the brain and spinal cord was significantly elevated [29].These findings further support the role of USP25 in regulating inflammatory responses. Additionally, Zhong et al. reported that overexpression of USP25 inhibits IL-17-triggered signaling and inflammation by interacting with TRAF5 and TRAF6 in helper lymphocyte-mediated immune responses [33]. This suggests that USP25 may function as a negative regulator of inflammatory signaling pathways triggered by cytokines such as IL-17. While the role of USP25 has been extensively studied in various immune-related contexts, its function in nephritis remains unexplored. Our data provides the first evidence for a protective role of USP25 in anti-GBM GN. USP25-deficient mice exhibited more severe glomerulonephritis pathology compared to wild-type mice. The immune response in anti-GBM GN is primarily mediated by Th1 and Th17 cells, while Th2 cells exert a protective effect [34]. Our results demonstrate that USP25 deficiency leads to increased infiltration of Th1 and Th17 cells into the kidney, as well as elevated expression of the Th17-specific transcription factor RORγt. This suggests that USP25 inhibits the differentiation of CD4+ T cells into Th17 cells, thereby limiting immune activation and renal damage in anti-GBM GN.

The apparent contradiction in the effect of USP25 on Th1 cell differentiation observed *in vivo* and *in vitro* is intriguing. *In vivo*, knockout of USP25 leads to an increase in both Th1 and Th17 cells in the mouse spleen, while USP25 knockout has minimal impact on Th1 differentiation when studied *in vitro*. We hypothesize that *in vivo* conditions are more complex, with dendritic cells producing IFN-γ and IL-12, which stimulate primitive T cells to differentiate into Th1 cells during the early stages. Subsequently, these differentiated Th1 cells themselves secrete a large amount of IFN-γ, creating a positive feedback loop that can amplify Th1 cell differentiation. However, this regulatory loop does not exist *in vitro* settings. Furthermore, knockout of USP25 *in vivo* enhances the immune response of mice, possibly leading to higher levels of Th1-stimulating factors, thus promoting Th1 cell differentiation. In contrast, *in vitro* experiments already include sufficient inflammatory factors to stimulate Th1 cell differentiation, and these conditions are not affected by USP25 knockout. These observations underscore the importance of considering the complex interactions and feedback loops that occur *in vivo* when studying the immune cell differentiation and function.

The regulation of USP25 and its expression changes during immune responses remain enigmatic. Current studies on USP25 are limited, despite its known roles in controlling various signaling pathways. Although there have been reports on the up-regulation of USP25 expression levels following RNA and DNA virus infections [33], LPS treatment [35], and in Down syndrome patients [36], the precise regulation of USP25 protein remains unclear. In the context of anti-GBM GN, our experimental results indicate that USP25 gene expression is up-regulated during the onset and progression of the disease, positively correlating with Th17 cell levels. To further investigate the association between USP25 expression and glomerulonephritis, we examined USP25 levels in *Fas^lpr^* mice, which exhibit autoimmune abnormalities resembling human systemic lupus erythematosus. These mice develop immune complex glomerulonephritis (type II CGN) due to the loss of the *Fas* gene. Consistent with previous studies [37], we found that the renal immune response and Th17 cell levels in *Fas^lpr^* mice increased before 19 weeks of age, paralleling the observed increase in USP25 expression. *In vitro* experiments also confirmed an increase in USP25 expression following Th17 cell differentiation. Based on these findings, we hypothesize that during anti-GBM GN, USP25 expression rises as Th17 cells differentiate, and USP25 negatively regulates Th17 cell differentiation. However, it’s noteworthy that this negative feedback mechanism is insufficient to halt TH17 cell differentiation and its inflammatory response formation. Otherwise, the kidneys of anti-GBM GN mice and *Fas^lpr^* mice would presumably achieve self-repair. Future studies are needed to elucidate the precise regulatory mechanisms of USP25, its interaction with other signaling pathways, and the molecular basis for its negative feedback on TH17 cell differentiation. Such insights could lead to new therapeutic strategies for targeting USP25 in inflammatory diseases like glomerulonephritis.

In conclusion, our findings reveal that USP25 exhibits a negative feedback modulation on Th17 differentiation and attenuates anti-GBM GN. Based on these observations, USP25 holds promise as a novel therapeutic target for mitigating the progression of anti-GBM GN.

Our study has certain limitations that merit attention. Firstly, while our research has demonstrated that USP25 can down-regulate the level of RORγt, the precise molecular mechanism underlying USP25’s regulation of Th17 differentiation remains incompletely understood. Future experiments will focus on further elucidating the interaction between USP25 and RORγt to extend the conclusions drawn from this study. Secondly, our study exclusively utilized USP25-deficient mice. The employment of siRNA techniques to knockdown USP25 expression in primary culture cells would have strengthened our findings, providing additional validation and credibility. However, due to funding constraints, the scale of our experiments, and the number of experimental animals used, we were unable to incorporate siRNA techniques into our study. Finally, this study primarily focused on the relationship between USP25 and Th17 cells in the context of anti-GBM GN. While Th17 cells play a central role, other inflammatory cells such as Th1 cells and neutrophils also contribute significantly to the pathogenesis of anti-GBM GN. Future projects should aim to explore the association between USP25 and these additional inflammatory cell types, thereby enriching the field of research in this area.

## Supplementary Material

Supplemental Material

## Data Availability

The datasets used and/or analyzed during the current study are available from the corresponding author upon reasonable request.
