# Supplementary material for: USP25 attenuates anti-GBM nephritis in mice by negative feedback regulation of Th17 cell differentiation
Source: Ren Fail. 2024 Apr 14;46(1):2338932. doi: 10.1080/0886022X.2024.2338932 (PMC11018034; doi:10.1080/0886022X.2024.2338932)
Supplement: Supplemental Material [file IRNF_A_2338932_SM0297.pdf]

USP25 Attenuates Anti-GBM Nephritis in Mice by Negative Feedback Regulation of Th17 Cell Differentiation

Ranran Xu[1], Fei Huang[2], Qingquan Liu[3], Yongman Lv[3, 4], Liu Hu[4], Qian Zhang[3]

1 Department of Geriatrics, Tongji Hospital, Tongji Medical College, Huazhong University of Science and Technology, 1095 Jie Fang Avenue, Hankou, Wuhan, 430030, P.R. China

2 Department of General Medicine, Tongji Hospital, Tongji Medical College, Huazhong University of Science and Technology, 1095 Jie Fang Avenue, Hankou, Wuhan, 430030, P.R. China

3 Department of Nephrology, Tongji Hospital, Tongji Medical College, Huazhong University of Science and Technology, 1095 Jie Fang Avenue, Hankou, Wuhan, 430030, P.R. China

4 Health Management Centre, Tongji Hospital, Tongji Medical College, Huazhong University of Science and Technology, 1095 Jie Fang Avenue, Hankou, Wuhan, 430030, P.R. China

The primers of Real-time PCR used as follows:

M-GAPDH-F CAGGAGAGTGTTTCCTCGTCC,

M-GAPDH-R TTCCCATTCTCGGCCTTGAC;

M-RORγt-F TGCAGTGCCCCAGAGGTACCATATG,

M-RORγt-R, CGCTCCCACATCTCCCACATTGAC;

M-FOXP3-F, GCCACAACCTGAGCCTGCACAAGT,

M-FOXP3-R, GGGGCGTTGGCTCCTCTTCTTG;

M-Tbx21-F, CGGGGTTGGAGGTGTCTGGGAA,

M-Tbx21-R, CGGCCACGGTGAAGGACAGGA;

M-Gata3-F, TCGGCCAGGCAAGATGAGAAAGAGT,

M-Gata3-R, CGGGCACATAGGGCGGATAGGT.

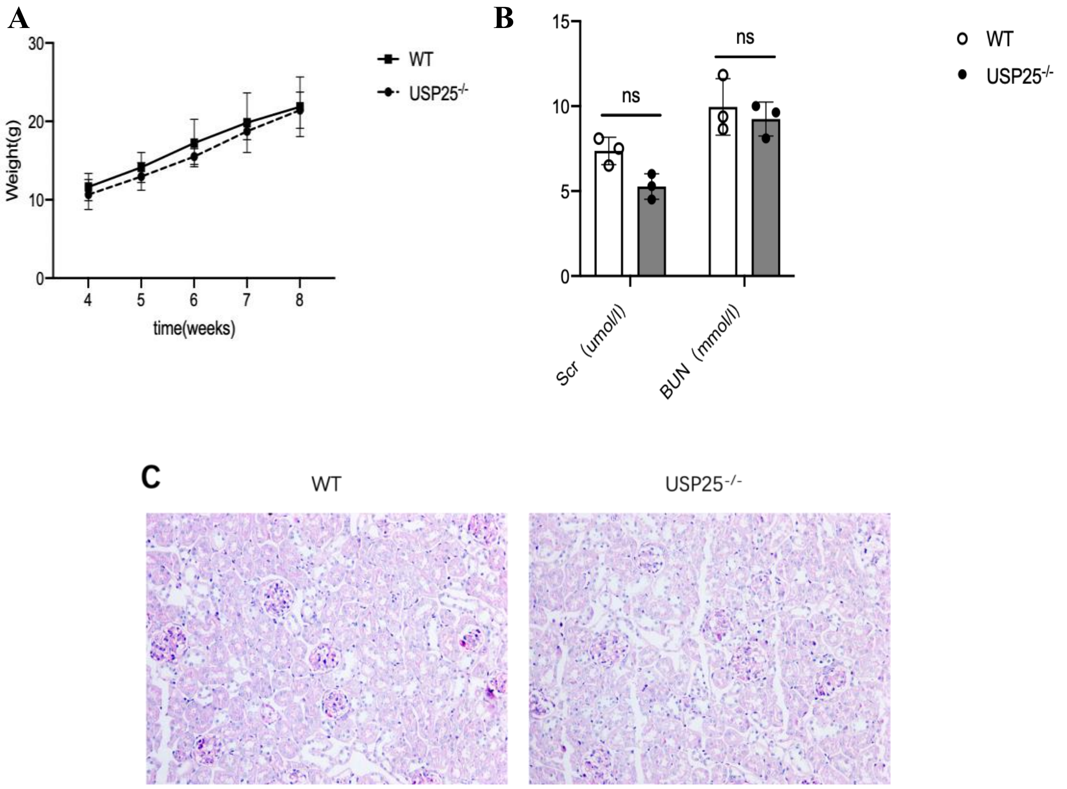

Fig.S1. Weight (A), renal function (B) and renal tissue (C) of WT mice and USP25$^{-/-}$ mice. WT: wild type; Bars represent means ± SEM; n = 5/group.

A CD4^{+}IFN-γ^{+} cells(%)

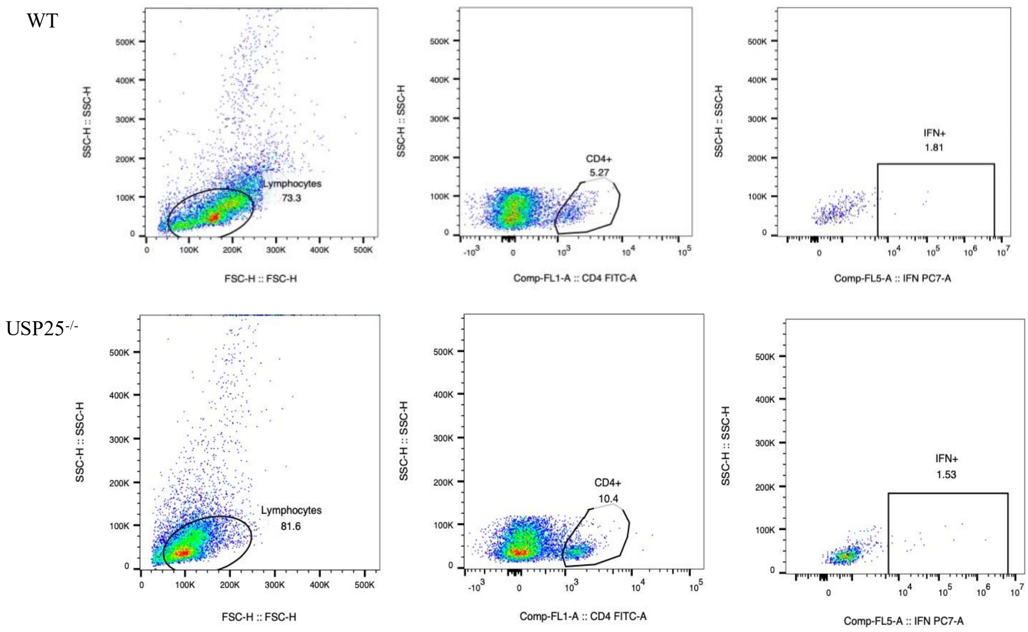

B CD4+IL-4+ cells(%)

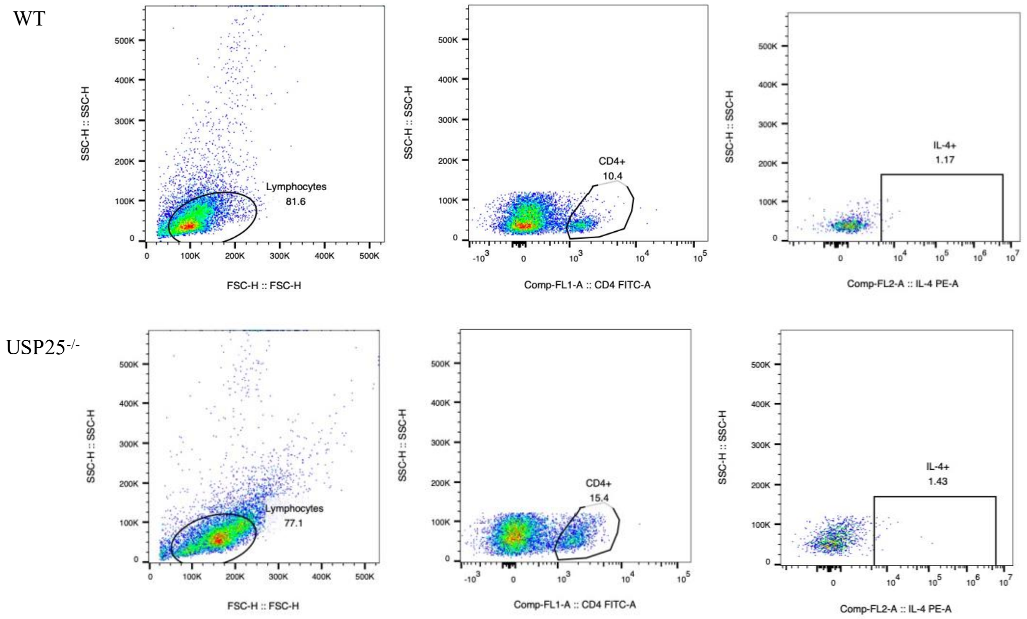

C CD4+IL-17A+ cells(%)

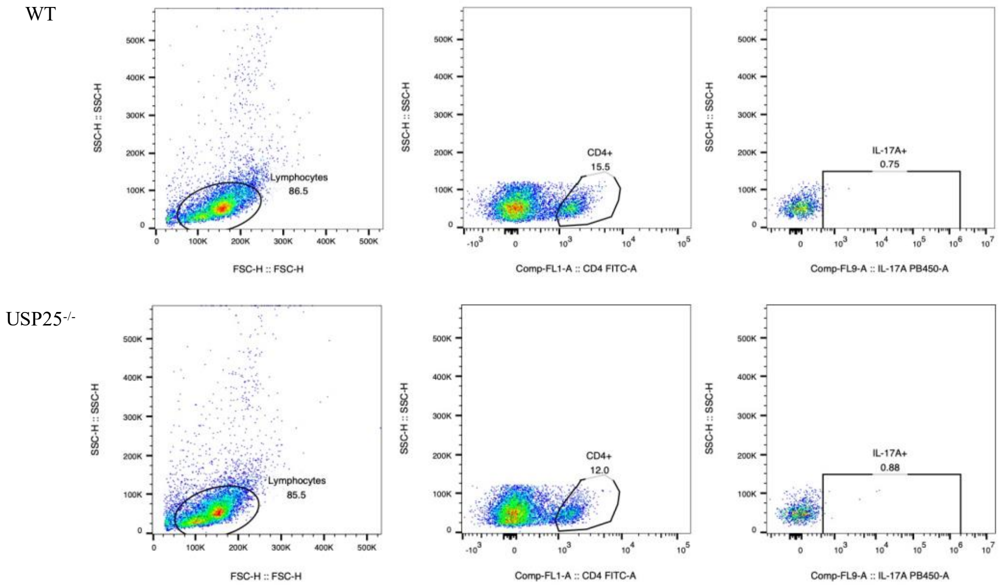

Fig.S2. The proportions of CD4+ T subsets (Th1 cells, Th2 cells, Th17 cells) in spleen of WT mice and USP25$^{-/-}$ mice.

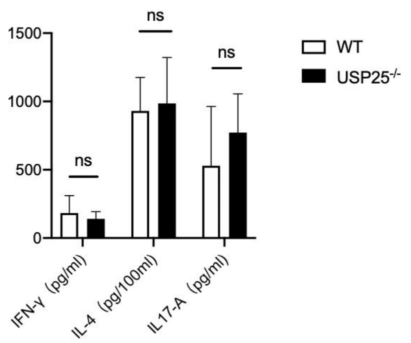

Fig.S3. Levels of inflammatory cytokines in blood of WT mice and USP25$^{-/-}$ mice.

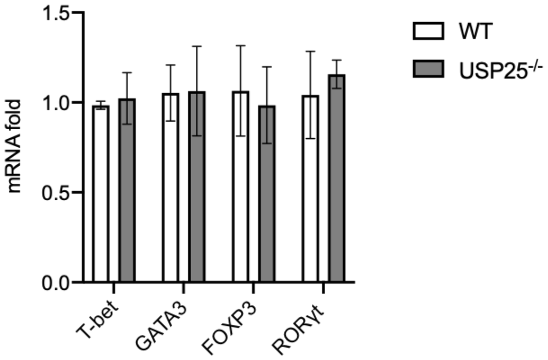

Fig.S4. Expression of helper lymphocyte cell transcription factors in kidney tissues of WT mice and USP25$^{-/-}$ mice.
